# Suspected Groove Pancreatitis Complicated by Anastomotic Stricture and Recurrent Intrahepatic Lithiasis: A Case Report

**DOI:** 10.7759/cureus.116777

**Published:** 2026-09-23

**Authors:** Maria Isabel de Sampaio Rabello, Natália Borges, Maria Paula Ribeiro Dantas Bezerra, Julia Angelim de Freitas Cardoso, Lawani Rigopoulos

**Affiliations:** 1 Gastroenterology, Hospital Israelita Albert Einstein, São Paulo, BRA; 2 Internal Medicine, Universidade Católica de Pernambuco, Recife, BRA; 3 Gastroenterology, Hospital das Clínicas da Faculdade de Medicina da Universidade de São Paulo, São Paulo, BRA; 4 Gastroenterology, Irmandade da Santa Casa de Misericórdia de São Paulo, São Paulo, BRA

**Keywords:** biliary-enteric stricture, cholangioplasty, cholangitis, groove pancreatitis, intrahepatic lithiasis, ptbd

## Abstract

Groove pancreatitis is a rare form of chronic pancreatitis involving the pancreatoduodenal groove and may mimic pancreatic or periampullary malignancy, creating substantial diagnostic uncertainty. We report a 67-year-old man with a history of alcohol use, a 10-pack-year smoking history, chronic pancreatitis, and suspected groove pancreatitis who underwent biliodigestive diversion, cholecystectomy, and pancreatic biopsy in 2017; malignancy was ultimately excluded. Several years later, he developed recurrent cholangitis associated with a biliary-enteric anastomotic stricture and progressive intrahepatic lithiasis. Imaging demonstrated persistent left intrahepatic ductal dilatation and anastomotic narrowing. Management consisted of percutaneous transhepatic biliary drainage, repeated drain exchanges, and serial cholangioplasties, with partial reduction of stone burden despite persistent stricture. This case illustrates the challenges of long-term management of late biliary complications after pancreatobiliary surgery and the potential role of serial minimally invasive interventions in maintaining biliary drainage and deferring surgical reintervention.

## Introduction

Groove pancreatitis is a rare form of chronic pancreatitis involving the anatomical space between the pancreatic head, the duodenum, and the common bile duct [1,2]. It predominantly affects middle-aged men and is strongly associated with alcohol consumption and tobacco smoking [1,2]. Pathologically, it is characterized by fibroinflammatory changes centered in the pancreaticoduodenal groove, often accompanied by duodenal wall thickening, cystic changes, and Brunner gland hyperplasia [1]. These changes may produce a mass-like appearance, and the clinical and imaging features may overlap with pancreatic adenocarcinoma, periampullary malignancy, and other focal inflammatory pancreatic processes, often creating substantial diagnostic uncertainty [3,4]. In selected cases, surgical intervention may therefore be required when malignancy cannot be confidently excluded or when obstructive symptoms persist.

Management strategies range from conservative and endoscopic approaches to definitive surgery, depending on symptom severity, anatomical changes, and suspicion of neoplasia [3]. Surgical treatment is frequently required in complex or refractory cases and is associated with high rates of symptom relief, particularly in the presence of persistent obstruction or unresolved diagnostic ambiguity [2]. However, biliary reconstruction may be followed by late complications, including benign biliary-enteric anastomotic strictures, which can impair biliary drainage and predispose to recurrent cholangitis [5,6].

Chronic biliary stasis may also promote recurrent intrahepatic lithiasis, further complicating long-term management. Persistent obstruction, altered biliary flow, and recurrent infection are key drivers of hepatolithiasis recurrence and often necessitate repeated interventional procedures rather than a single definitive treatment [7,8].

We report a case of suspected groove pancreatitis treated surgically, followed by a prolonged and complex biliary course characterized by biliary-enteric anastomotic stricture, recurrent cholangitis, intrahepatic lithiasis, and the need for serial percutaneous interventions. The principal educational focus of this case is the long-term management of these late biliary complications rather than the initial pancreatic diagnosis alone. This case highlights the challenges of sustained management after biliary reconstruction and the role of minimally invasive strategies in maintaining biliary drainage and deferring repeat surgical revision in selected patients.

## Case presentation

A 67-year-old man with a history of alcohol use, a 10-pack-year smoking history, chronic pancreatitis, and suspected groove pancreatitis underwent biliodigestive bypass, cholecystectomy, and pancreatic biopsy in 2017; malignancy was excluded. His medical history also included insulin-dependent diabetes mellitus, systemic arterial hypertension, and prior cholelithiasis.

In 2023, he developed recurrent cholangitis secondary to biliary-enteric anastomotic stricture. Given the obstructive biliary pattern in the setting of prior biliodigestive reconstruction, percutaneous transhepatic biliary drainage (PTBD) was performed, followed by scheduled drain exchanges during follow-up (September 2023, February 2024, August 2024, and January 2025).

Magnetic resonance cholangiopancreatography (MRCP) demonstrated persistent dilation of the left intrahepatic bile ducts, with associated intrahepatic lithiasis and anastomotic narrowing (Figure 1). A focal hepatic lesion suggestive of a developing cholangiolytic abscess was also identified (Figure 2). Given the ongoing obstructive pattern, serial cholangioplasties were subsequently performed (Figure 3), resulting in partial reduction of stone burden, although the stricture persisted.

**Figure 1:**
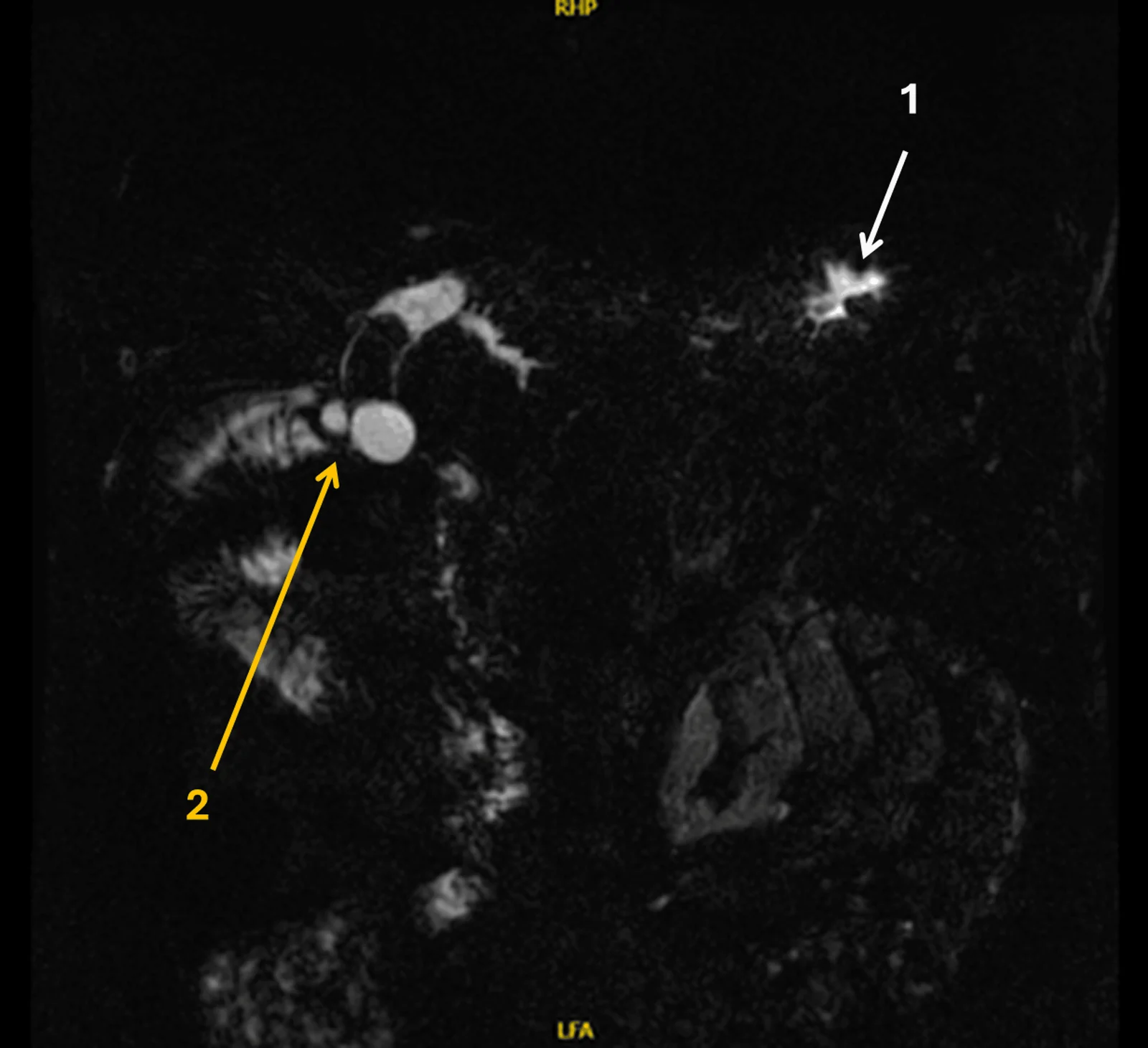
Initial MRCP obtained during evaluation of the biliary complication, demonstrating left-predominant intrahepatic biliary dilation (1) with multiple intraductal filling defects consistent with intrahepatic lithiasis. A large obstructing calculus (2) is identified at the biliodigestive anastomosis. MRCP: magnetic resonance cholangiopancreatography

**Figure 2:**
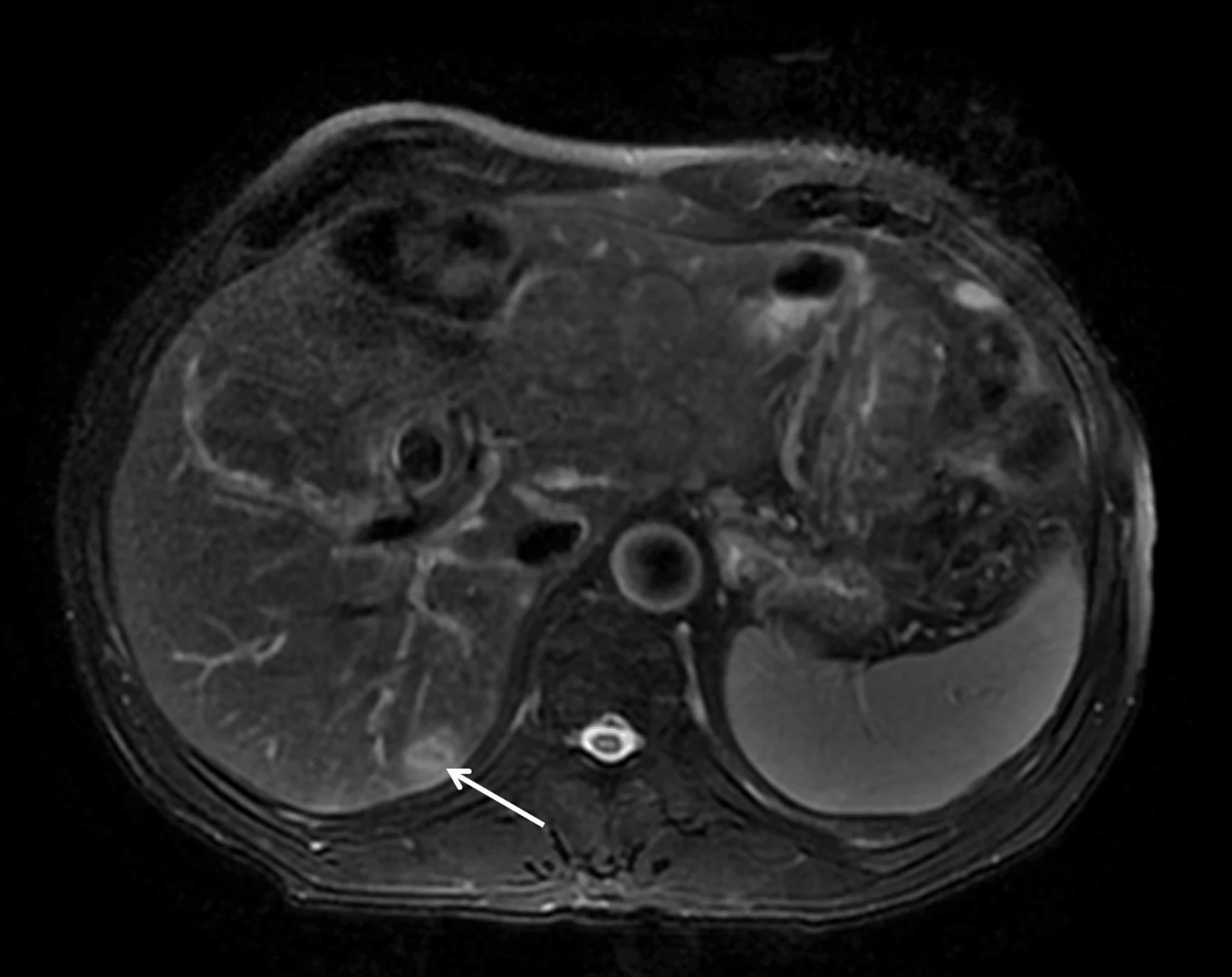
Axial T2-weighted MRI from the initial imaging evaluation demonstrating a focal hyperintense lesion in the right hepatic lobe. The arrow indicates the lesion, which is suggestive of a developing cholangiolytic abscess.

**Figure 3:**
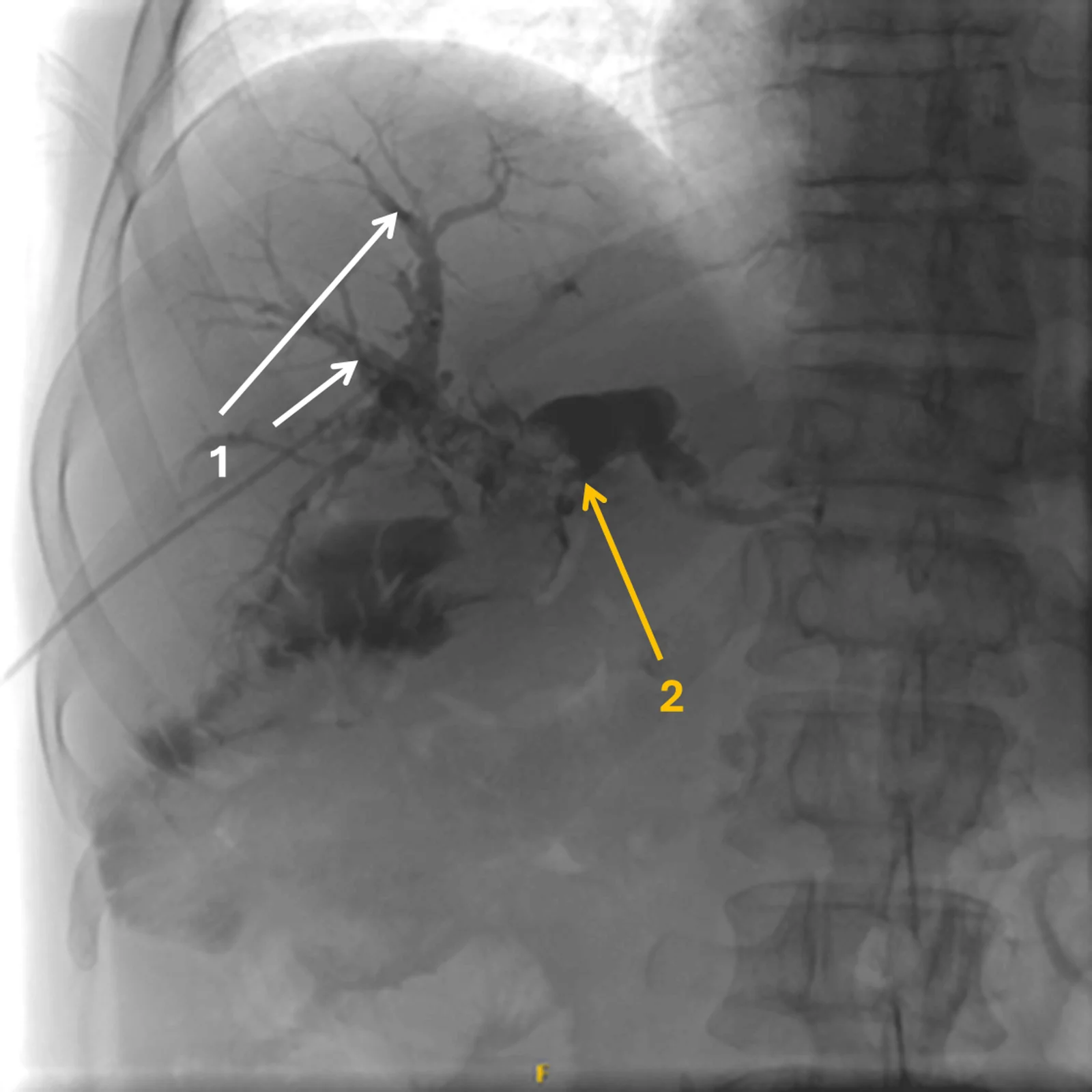
Percutaneous transhepatic cholangiography obtained during subsequent interventional management, demonstrating intrahepatic filling defects (1) consistent with stones and a biliodigestive anastomotic stricture. Balloon cholangioplasty (2) is performed at the anastomosis.

Interval imaging demonstrated partial improvement in biliary dilatation, with persistent left-sided involvement and residual intraductal defects (Figure 4).

**Figure 4:**
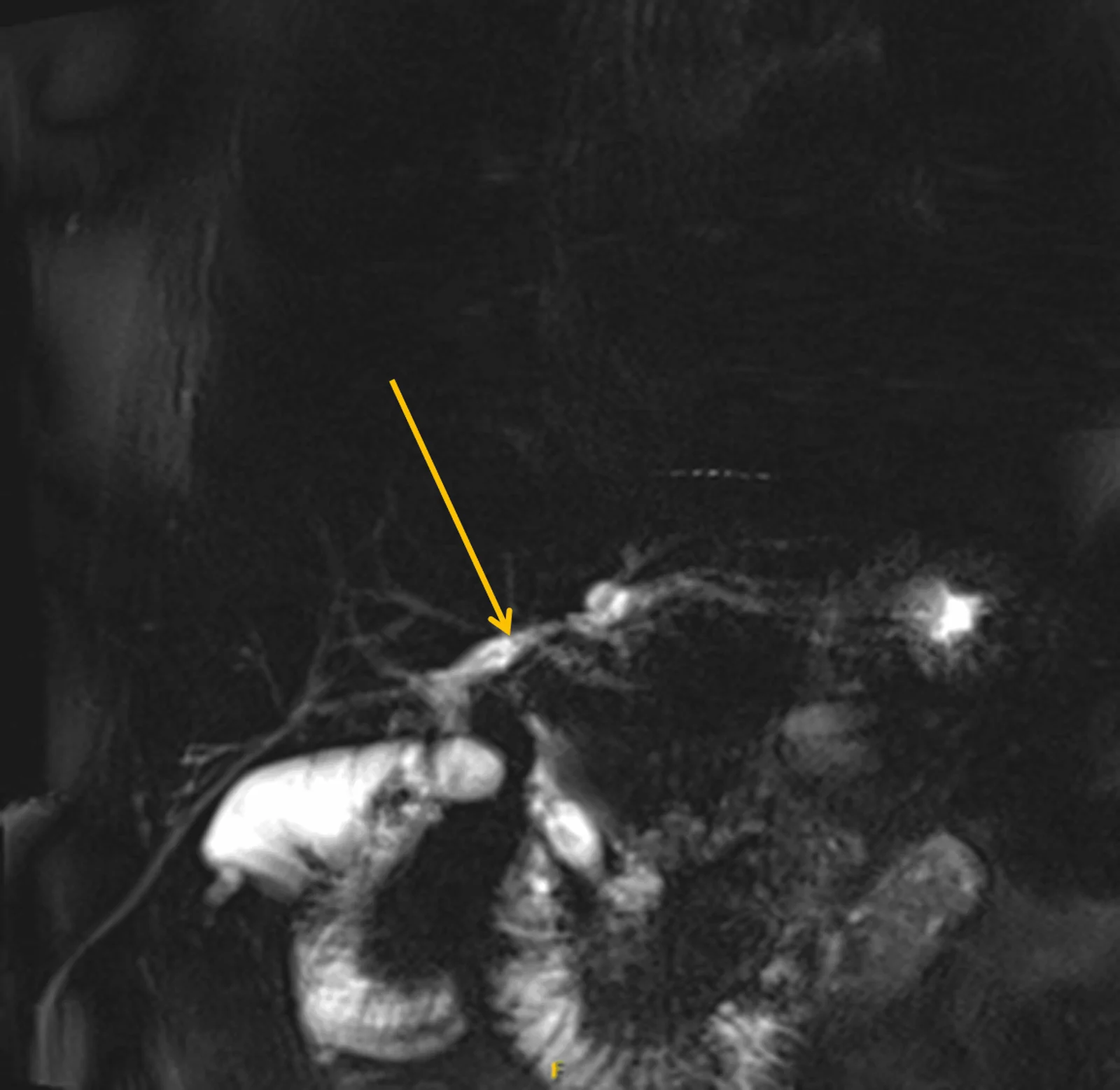
Follow-up MRCP demonstrating persistent left-predominant intrahepatic biliary dilatation with residual intraductal filling defects. The arrow highlights the persistent left intrahepatic biliary dilatation. MRCP: magnetic resonance cholangiopancreatography

At the latest documented follow-up, the patient remained under outpatient follow-up with interventional radiology, with ongoing PTBD management and imaging surveillance. Follow-up cholangiography demonstrated persistent intrahepatic lithiasis, predominantly in the left biliary system, with residual anastomotic stricture despite repeated interventions (Figure 5).

**Figure 5:**
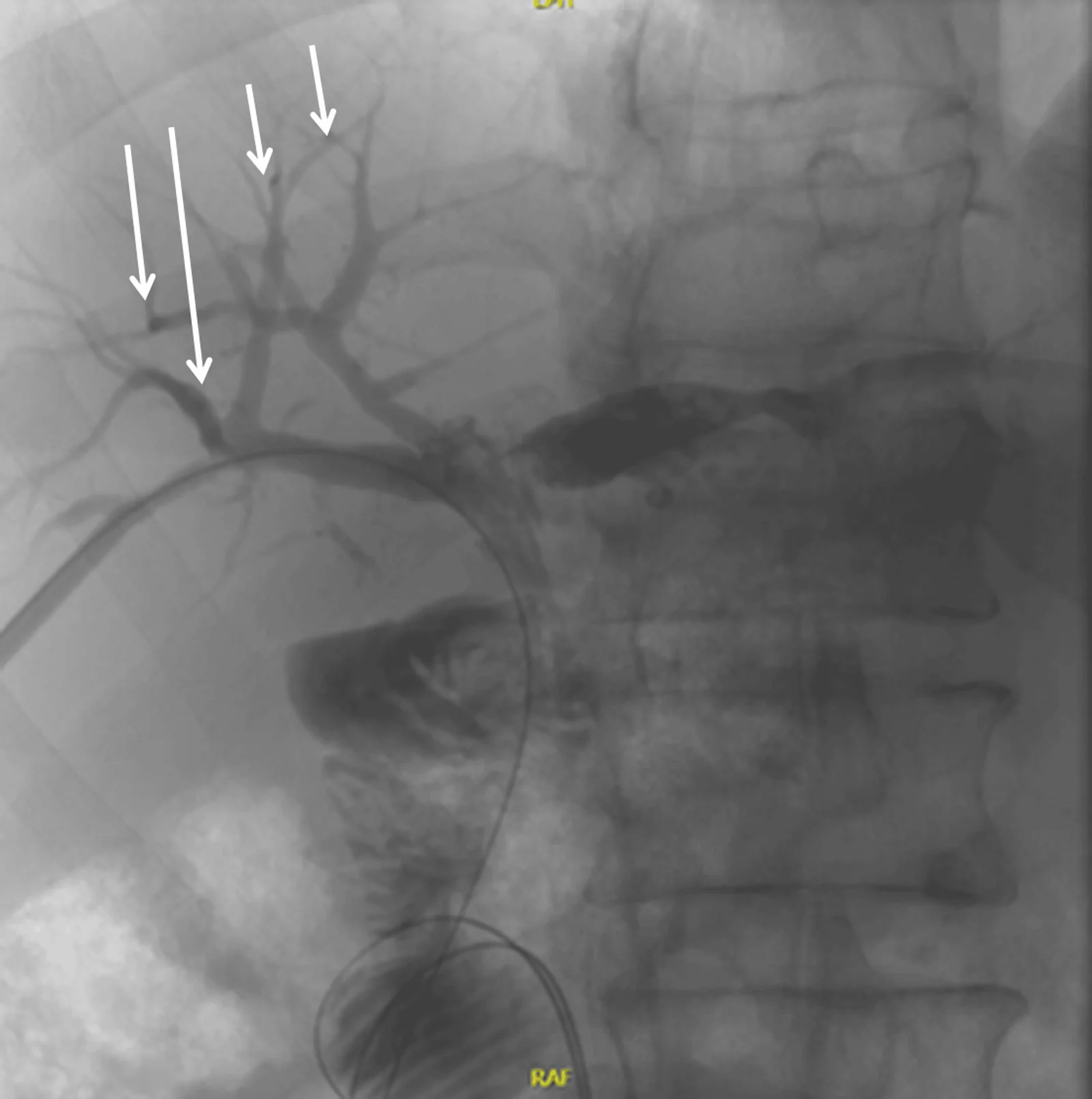
Follow-up percutaneous cholangiography demonstrating persistent intrahepatic filling defects, predominantly in the left biliary system, with residual anastomotic stricture. The arrows indicate the persistent intrahepatic filling defects. Bilateral biliary drains are in place.

A summary of the major clinical, imaging, and interventional events is presented in Table 1.

**Table 1:** Summary of the patient's clinical course and interventional management MRCP: magnetic resonance cholangiopancreatography

Time period	Clinical findings	Intervention
2017	Chronic pancreatitis with suspected groove pancreatitis; malignancy considered in the differential diagnosis	Biliodigestive bypass, cholecystectomy, and pancreatic biopsy; malignancy ultimately excluded
2023	Recurrent cholangitis associated with biliary-enteric anastomotic stricture and obstructive biliary findings	Percutaneous transhepatic biliary drainage (PTBD) initiated
September 2023	Ongoing biliary drainage requirement	Scheduled biliary drain exchange
During subsequent follow-up	MRCP demonstrated persistent left-predominant intrahepatic biliary dilatation, intrahepatic lithiasis, anastomotic narrowing, and a focal hepatic lesion suggestive of a developing cholangiolytic abscess	Continued imaging surveillance and interventional management
February 2024	Ongoing biliary drainage requirement	Scheduled biliary drain exchange
During longitudinal interventional management	Persistent biliary obstruction and intrahepatic lithiasis	Serial cholangioplasties performed, with partial reduction in stone burden despite persistent anastomotic stricture
August 2024	Ongoing biliary drainage requirement	Scheduled biliary drain exchange
January 2025	Ongoing biliary drainage requirement	Scheduled biliary drain exchange
Latest documented follow-up	Persistent intrahepatic lithiasis, predominantly involving the left biliary system, with residual anastomotic stricture	Continued outpatient interventional radiology follow-up, PTBD management, bilateral biliary drains, and imaging surveillance

## Discussion

Groove pancreatitis is an uncommon and diagnostically challenging form of chronic pancreatitis involving the pancreatoduodenal groove. Its clinical and radiological features often overlap with pancreatic or periampullary malignancy, leading to diagnostic uncertainty and, in selected cases, surgical intervention [1,2,4]. Although conservative and endoscopic approaches are available, surgery is still required in a subset of patients, particularly when malignancy cannot be excluded or when obstructive symptoms persist [2,3].

In the present case, however, the primary clinical relevance lies not in the initial pancreatic diagnosis itself, but in the prolonged and complex biliary course that followed surgical management. This evolution was characterized by biliary-enteric anastomotic stricture, recurrent cholangitis, and intrahepatic lithiasis, ultimately requiring repeated interventional procedures.

Benign biliary-enteric anastomotic strictures are well-recognized late complications of biliary reconstruction and represent an important source of long-term morbidity [5,6]. A long-term series after hepaticojejunostomy reported an anastomotic stricture rate of approximately 13%, with delayed presentation and repeated radiological or surgical interventions in affected patients [9]. Their clinical impact extends beyond impaired bile flow, as persistent biliary obstruction promotes a cycle of stasis, inflammation, and recurrent infection [5,10]. Percutaneous treatment may provide durable control in a substantial proportion of patients, although prolonged drainage or eventual surgical revision may still be required in refractory cases [11]. In this patient, the development of cholangitis several years after biliodigestive diversion underscores the delayed and progressive nature of this complication.

The persistence of the stricture despite sequential interventions suggests that these lesions may behave as chronic and refractory conditions rather than isolated technical failures. This is particularly relevant in patients with complex pancreatobiliary anatomy or longstanding inflammatory disease, in whom complete and durable resolution may be difficult to achieve [6,12,13].

The association between anastomotic stricture and recurrent intrahepatic lithiasis is both pathophysiologically plausible and clinically relevant. Altered biliary flow, chronic stasis, and recurrent bacterial contamination are key mechanisms driving the development and recurrence of hepatolithiasis [7,8]. Once established, intrahepatic stones may sustain a self-perpetuating cycle of obstruction, cholangitis, and further stone formation, increasing morbidity and the need for repeated interventions [7,8,14]. In the present case, persistent left intrahepatic ductal dilation associated with calculi and anastomotic narrowing reflects this chronic obstructive-inflammatory process and illustrates how a postoperative biliary complication may evolve into a progressive, longitudinal disease rather than remain an isolated event.

Another important aspect is the role of longitudinal imaging and procedural reassessment. In complex benign biliary disease, serial imaging is not merely confirmatory but essential for monitoring ductal dilation, stone burden, and treatment response over time [8]. In this case, magnetic resonance imaging provided a comprehensive anatomical assessment, demonstrating persistent left-sided biliary dilation, intrahepatic lithiasis, and ongoing anastomotic stricture. These findings supported a staged interventional approach. This longitudinal radiologic perspective is particularly valuable when complete anatomical resolution of the stricture is not achieved, and management instead focuses on maintaining biliary drainage, monitoring stone burden, and guiding the need for repeated interventions.

From a therapeutic standpoint, the patient’s prior biliodigestive reconstruction, altered postoperative anatomy, chronic pancreatobiliary disease, and medical comorbidities provided a clinical context favoring continuation of a minimally invasive strategy over repeat surgical revision. PTBD is a well-established option for benign biliary strictures, especially in patients with altered postoperative anatomy, and may be combined with balloon dilatation and repeated catheter exchanges as part of a stepwise approach [5,6,12-15]. Endoscopic treatment, particularly balloon enteroscopy-assisted endoscopic retrograde cholangiopancreatography (ERCP), may also provide effective access to biliary-enteric anastomotic strictures, although technical success depends on the ability to reach and traverse the anastomosis in surgically altered anatomy.

In a retrospective series of hepaticojejunostomy anastomotic strictures, double-balloon enteroscopy-assisted ERCP (DBE-ERCP) alone achieved clinical success in 49% of patients, while PTBD was required in 49%, including combined endoscopic and percutaneous therapy in 22% [16]. More recently, a comparative study of hepatolithiasis after hepaticojejunostomy found similar complete stone clearance with balloon-assisted enteroscopic and percutaneous approaches (85.7% and 90.2%, respectively), with no significant difference in stone recurrence, although hospitalization was shorter with endoscopic treatment [17]. These findings support individualized selection of the therapeutic route rather than a universal preference for either approach, with percutaneous treatment remaining particularly relevant when endoscopic access is limited or insufficient.

Percutaneous treatment is not uniformly durable, and poorer outcomes have been associated with more complex disease patterns, particularly multiple biliary strictures and the need for repeated treatment courses [18]. Such features reinforce the importance of longitudinal reassessment and individualized escalation of therapy in persistent or recurrent strictures [15,18]. In this context, treatment success should not be defined solely by complete stricture resolution. Instead, clinically meaningful outcomes include sustained biliary decompression and progressive reduction in stone burden [11-15,18]. In the present case, although the stricture persisted, serial cholangioplasties were associated with a partial reduction in lithiasis burden, while continued interventional follow-up and PTBD management were maintained to preserve biliary drainage.

This case highlights the importance of individualized and multidisciplinary management in complex postoperative biliary disease. In patients with chronic pancreatobiliary disorders, prior reconstructive surgery, and recurrent obstruction, therapeutic decisions must balance the potential for anatomical correction against procedural risk, durability of results, and overall clinical stability [5,6,8,15]. Rather than suggesting superiority of percutaneous approaches over surgical revision, this report supports a more nuanced perspective: in selected patients with prior biliary reconstruction, altered postoperative anatomy, and chronic recurrent obstruction, serial minimally invasive interventions may represent a pragmatic strategy for long-term biliary management when repeat surgery is less desirable [11,16-18]. This is particularly relevant in prolonged clinical courses such as the one described, where maintaining biliary drainage and ensuring close multidisciplinary follow-up become primary therapeutic goals.

## Conclusions

This case highlights the complex long-term biliary course that may follow surgical management of suspected groove pancreatitis, characterized by biliary-enteric anastomotic stricture and recurrent intrahepatic lithiasis requiring repeated interventions and sustained follow-up.

Serial imaging, cholangioplasty, and close coordination with interventional radiology supported continued biliary drainage and were associated with a partial reduction in stone burden despite persistent anastomotic stricture. These findings underscore the importance of individualized, multidisciplinary management in complex post-surgical biliary obstruction. In selected patients, serial minimally invasive interventions may provide a pragmatic strategy for maintaining biliary drainage and deferring repeat surgical revision when reoperation is less desirable.
